# Polarization- and wavelength-agnostic nanophotonic beam splitter

**DOI:** 10.1038/s41598-019-40497-7

**Published:** 2019-03-05

**Authors:** David González-Andrade, Christian Lafforgue, Elena Durán-Valdeiglesias, Xavier Le Roux, Mathias Berciano, Eric Cassan, Delphine Marris-Morini, Aitor V. Velasco, Pavel Cheben, Laurent Vivien, Carlos Alonso-Ramos

**Affiliations:** 1 Instituto de Óptica Daza de Valdés, Consejo Superior de Investigaciones Científicas (CSIC), Madrid, 28006 Spain; 2 Centre de Nanosciences et de Nanotechnologies, CNRS, Université Paris-Sud, Université Paris-Saclay, C2N, Orsay, 91405 France; 3 École Normale Supérieure Paris-Saclay, Université Paris-Saclay, Cachan, 94230 France; 4 National Research Council Canada, 1200 Montreal Road, Ottawa, Ontario K1A0R5 Canada

## Abstract

High-performance optical beam splitters are of fundamental importance for the development of advanced silicon photonics integrated circuits. However, due to the high refractive index contrast of silicon-on-insulator platforms, state-of-the-art nanophotonic splitters are hampered by trade-offs in bandwidth, polarization dependence and sensitivity to fabrication errors. Here, we present a new strategy that exploits modal engineering in slotted waveguides to overcome these limitations, enabling ultra-broadband polarization-insensitive optical power splitters with relaxed fabrication tolerances. The proposed splitter design relies on a single-mode slot waveguide that is gradually transformed into two strip waveguides by a symmetric taper, yielding equal power splitting. Based on this concept, we experimentally demonstrate −3 ± 0.5 dB polarization-independent transmission for an unprecedented 390 nm bandwidth (1260–1650 nm), even in the presence of waveguide width deviations as large as ±25 nm.

## Introduction

Silicon-on-insulator (SOI) platforms are becoming established as an enabling technology for next-generation photonic circuits for a wide range of applications, including telecom and datacom applications^1–3^, radio-over-fibre systems^4,5^, bio-sensing^6,7^, LIDAR^8^ and absorption spectroscopy^9,10^, to name a few. Such applications would benefit from the low-cost and large-volume fabrication offered by existing CMOS facilities as well as from the high density of integration enabled by the high refractive index contrast of SOI platforms. Furthermore, the high modal confinement of Si wire waveguides provides strong light-matter interactions with great potential for exploitation in opto-electronics, sensing and non-linear optical devices^11,12^. However, the index contrast and modal confinement of SOI platforms also pose important challenges for the realization of high-performance SOI circuits, including a strong sensitivity to small geometric deviations, strong modal dispersion, and large birefringence between the transverse electric (TE) and transverse magnetic (TM) modes. Hence, SOI circuits typically operate in a single polarization state, within a limited bandwidth and with tight fabrication tolerances. Nevertheless, polarization-independent devices have emerged showing very similar performance for both TE and TM polarizations^13^. High-performance SOI fibre-chip interfaces have been demonstrated to yield broadband and high-efficiency coupling with negligible polarization dependence and relaxed fabrication tolerances^14–16^, but such performance enhancements are still sought after in other essential SOI components. Specifically, beam splitters are particularly sensitive to the effects related to high index contrast and tight mode confinement in Si wires and would greatly benefit from achieving ultra-broadband dual-polarization operation.

Over the past few years, several beam splitters with different advantages and limitations have been proposed. Directional couplers (DCs) are based on two parallel waveguides separated by a gap, enabling straightforward tuning of the power-splitting ratio by adequately selecting the coupling length. Due to their mode-beating-based operational principle, DCs are intrinsically narrowband in nature and suffer from a low tolerance to fabrication errors and a strong polarization dependence^17–19^. The constraints on fabrication tolerances have been alleviated by engineering the excitation of odd and even modes in shallow-etched^20^ and fully etched DCs^21–24^, but only single-polarization operation and dual-polarization operation over a limited bandwidth have been demonstrated. Silicon-bent^13^ and subwavelength-grating-assisted^25–28^ (SWG) DCs have recently been demonstrated to exhibit polarization-independent and ultra-broadband behaviour separately, but these approaches are still hampered by a high sensitivity to manufacturing deviations. Alternatively, symmetric Y-junctions or Y-branches have been demonstrated as theoretically lossless splitters^29–31^. However, their performance is significantly degraded in real fabrication scenarios due to the limited etching resolution when defining the abrupt discontinuity at the branch intersection. Consequently, the broad bandwidth and reduced polarization dependence are maintained, but a comparatively high insertion loss is exhibited. To overcome this limitation, several Y-branch variations^32–34^ have emerged, showing relatively broad bandwidths. Multimode interference couplers (MMIs)^35^ exploit the self-imaging (Talbot) effect to achieve low losses with improved fabrication tolerances but provide only limited bandwidth- and polarization-dependent behaviour. Sub-wavelength engineering has been applied to increase the operational bandwidths of MMIs^36,37^, but only in single-polarization operation. Other alternatives include inverse tapers^38^, star couplers^39^, adiabatic couplers^40^ and photonic crystal waveguides^41^, all of which are limited in terms of either operational bandwidth, fabrication requirements or design complexity. Experimental results of aforementioned beam splitters are summarized in Table 1, where it is observed that most of them operate in a single polarization state, usually TE. Only Halir *et al*. have experimentally demonstrated a device with a bandwidth broader than 300 nm^37^. Their subwavelength engineered MMI presents insertion loss and imbalance below 1 dB over a 325 nm wavelength range, in addition to a compact footprint of only 3.25 × 25.4 μm^2^, but operates only for TE polarization. On the contrary, polarization-insensitive beam splitters tend to increase the length of the device to attain low insertion loss and imbalance over a broad bandwidth. For example, the device proposed by Xu *et al*. exhibits a length longer than 120 μm and operates within a 100 nm bandwidth for both TE and TM polarizations simultaneously^23^, whereas the bandwidth is reduced to only 70 or 80 nm in other devices with more compact footprints.

**Table 1:** Experimental performance of state-of-the-art silicon beam splitters including insertion loss, imbalance, bandwidth, device length and polarization operation. Worst performance between TE and TM polarizations is considered for polarization-independent beam splitters. Note: values marked with an asterisk are extracted from either figures or splitting ratio data from the references.

Reference	Insertion loss	Imbalance	Bandwidth	Length	Polarization
Adiabatic splitter^20^	<0.31 dB	<0.2 dB	100 nm	300 μm	Single-polarization (TE)
SWG-assisted coupler^25^	<0.11 dB	<0.3 dB	185 nm	35 μm	
Y-junction^32^	<0.28 dB	<0.02 dB	80 nm	2 μm	
SWG MMI^37^	<1 dB	<1 dB	325 nm	25.4 μm	
Inverse tapers^38^	<0.4 dB	<0.68 dB	40 nm	12.5 μm	
Star coupler^39^	<1 dB	<1 dB	90 nm	0.75 μm	
Photonic crystal^41^	<0.25 dB	<0.58 dB	30 nm	20 μm	
Bent DC^13^	<1 dB	<0.87 dB*	80 nm	<50 μm	Polarization-independent (TE and TM)
Adiabatic coupler^23^	—	<0.7 dB	100 nm	185 μm	
Y-branch variation (tapers)^34^	<0.19 dB	<0.47 dB	70 nm	20 μm	
This work	<1 dB	<1 dB	390 nm	200 μm	

In this work, we propose and experimentally demonstrate a new beam splitter concept based on modal-engineered slotted waveguides. This design provides ultra-broadband and polarization-independent operation with relaxed fabrication tolerances. Our experimental results demonstrate a near-ideal transmission of −3 ± 0.5 dB in an unprecedented bandwidth of 390 nm for both the TE and TM polarizations.

## Results

**Figure 1:**
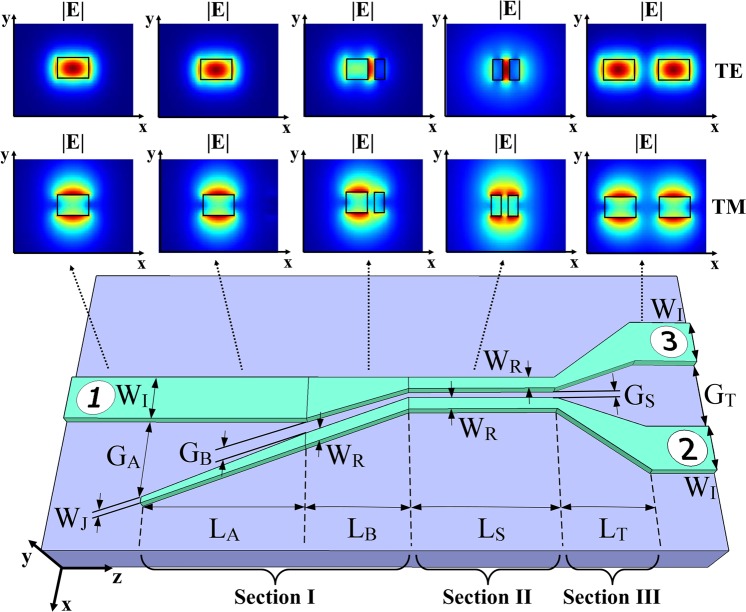
Device schematic and main design parameters of the broadband polarization-independent beam splitter. Electric field mode profiles at different sections are shown for both TE and TM polarization.

The proposed beam splitter, schematically shown in Fig. 1, relies on a single-mode slot waveguide that equally splits the injected power into two output strip waveguides in a wavelength- and polarization-agnostic fashion. Our device comprises three main sections: a strip-to-slot mode converter (section I), a slot waveguide (section II) and a slot-to-strip splitting transition (section III). The single-mode operation of the slot waveguide is of fundamental importance to our device because it mitigates any wavelength-dependent beating between different waveguide modes, which is the main phenomenon limiting the bandwidths of DCs and MMIs. The adiabatic strip-to-slot mode converter ensures the low-loss excitation of the fundamental (TE or TM) slot-waveguide mode^42,43^, while the symmetry of the slot-to-strip transition ensures equal power splitting between the two output waveguides, independent of the polarization and wavelength. Furthermore, this transition is robust against common under- and over-etching errors, as the latter do not break the structural symmetry, thus allowing the power-splitting ratio to be maintained. Finally, as the slot waveguide already includes a central gap, our transition circumvents any abrupt index discontinuity, minimizing back-reflections which are a major source of loss in conventional Y-junctions. Figure 1 also shows the electric field mode profile at each section of our proposed device, obtained via Finite-Difference Eigenmode (FDE) solver.

**Figure 2:**
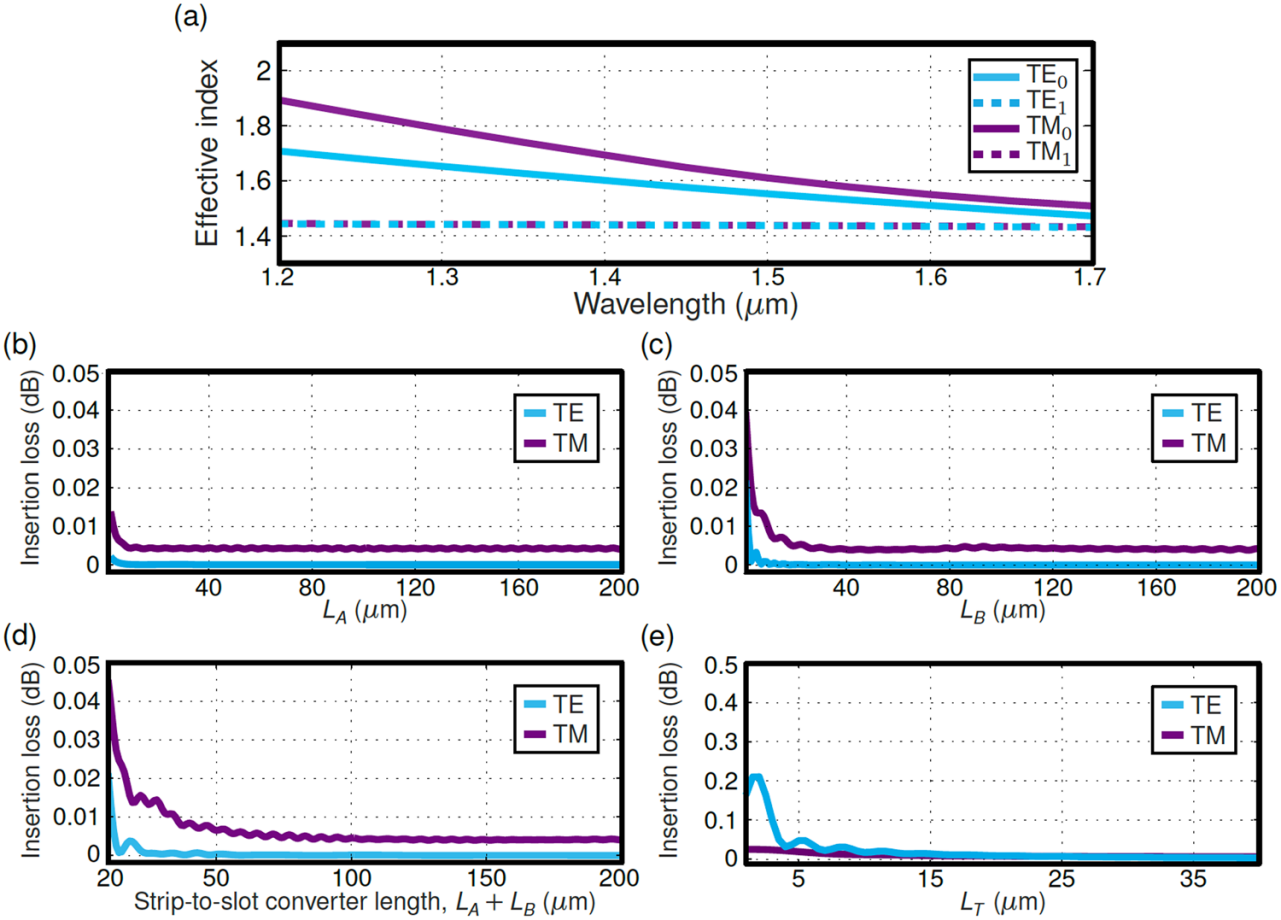
(**a**) Effective index as a function of the wavelength for the fundamental and first-order TE and TM slot waveguide modes. Slot waveguide with *W*_*R*_ = 150 nm and *G*_*S*_ = 100 nm (see Fig. 1). Higher order modes with effective index near ~1.45 are below the cut-off condition. Calculated insertion loss as a function of lengths *L*_*A*_ (**b**) and *L*_*B*_ (**c**), considered separately. (**d**) Insertion loss as a function of the overall strip-to-slot converter length (Fig. 1 section I) for the fundamental TE and TM modes. The lengths *L*_*A*_ and *L*_*B*_ are varied maintaining a 2:1 ratio. (**e**) Calculated insertion loss as a function of taper length *L*_*T*_ in section III. The design wavelength of 1.55 *µ*m is considered for insertion loss calculations in (**b**–**e**).

In our design 220-nm-thick and 450-nm-wide (*W*_*I*_) silicon-wires were used as the input and output strip waveguides. A buried silicon dioxide layer (BOX) and polymethyl methacrylate (PMMA) upper cladding were assumed, with refractive indexes *n*_*Si*_ = 3.476, *n*_*SiO*2_ = 1.444 and *n*_*PMMA*_ = 1.481, respectively, at the design wavelength of 1.55 *µ*m. First, we design the central slot waveguide in section II for single mode operation with no propagating higher order modes^44,45^. The slot waveguide comprises two narrow silicon waveguides, hereafter rails, separated by a low refractive index gap. We choose a rail width of *W*_*R*_ = 150 nm and a slot width of *G*_*S*_ = 100 nm, ensuring single-mode behaviour in the wavelength range between 1200 nm and 1700 nm (see Fig. 2(a)). Effective indexes of the modes supported by the slot waveguide in Fig. 2(a) were obtained with the FDE solver using E. D. Palik material database^46^ for material dispersion of silicon (Si) and silicon dioxide (SiO_2_). The strip-to-slot transition in the coupler (section I) is realized in two steps. In the first region of length *L*_*A*_, a narrow rail progressively approaches the strip waveguide, while in the second region of length *L*_*B*_, the strip waveguide and the narrow rail are combined into a slot waveguide. The rail at the beginning of the transition has a width of *W*_*J*_ = 100 nm and is separated from the strip waveguide by a gap of *G*_*A*_ = 850 nm, to preclude light coupling. After the first region of section I, the rail width is increased to *W*_*R*_ = 150 nm and the gap is reduced to *G*_*B*_ = *W*_*R*_ + *G*_*S*_ = 250 nm in order to adapt the gap size adiabatically from *G*_*A*_ to *G*_*S*_ along the entire section I. Figure 2(b,c) show the transition loss for both TE and TM modes as a function of the taper length *L*_*A*_ and *L*_*B*_ separately. Nevertheless, a ratio 2:1 is fixed for the lengths of tapers *L*_*A*_ and *L*_*B*_, yielding a constant slope with respect to the optical axis in the strip-to-slot transition (section I in Fig. 1). Calculated insertion loss for the strip-to-slot transition is below 0.005 dB for a total taper length (*L*_*A*_* + L*_*B*_) beyond 100 *µ*m (see Fig. 2(d)). For the fabrication of the device *L*_*A*_ = 80 *µ*m and *L*_*B*_ = 40 *µ*m were chosen. According to the simulations, a 99% coupling efficiency is obtained between each fundamental input mode and the slot mode of the same polarization. This is an acceptable value. However, fabrication imperfections in the tapers may result in unwanted and uncontrolled radiation that might be re-coupled in the output sections. Hence, the slot length in section II is set to *L*_*S*_ = 60 *µ*m in order to radiate away any residual power which is not coupled to the slot waveguide mode. Finally, the slot-to-strip splitting transition (section III) is implemented by two symmetric trapezoidal tapers that increase the rail width from *W*_*R*_ = 150 nm to *W*_*I*_ = 450 nm and the slot width from *G*_*S*_ = 100 nm to *G*_*T*_ = 350 nm. Insertion loss as a function of the taper length *L*_*T*_ is shown for both the TE and TM polarizations in Fig. 2(e). A transition length of *L*_*T*_ = 20 *µ*m was chosen for adiabatic behaviour. Note that in the fabricated devices, the final separation between both output strip waveguides, *G*_*T*_, is further increased to 1.75 *µ*m by means of conventional S-bends. The selected S-bends are cosine-shaped, with a fixed waveguide width of 450 nm and a length of 20 *µ*m.

**Figure 3:**
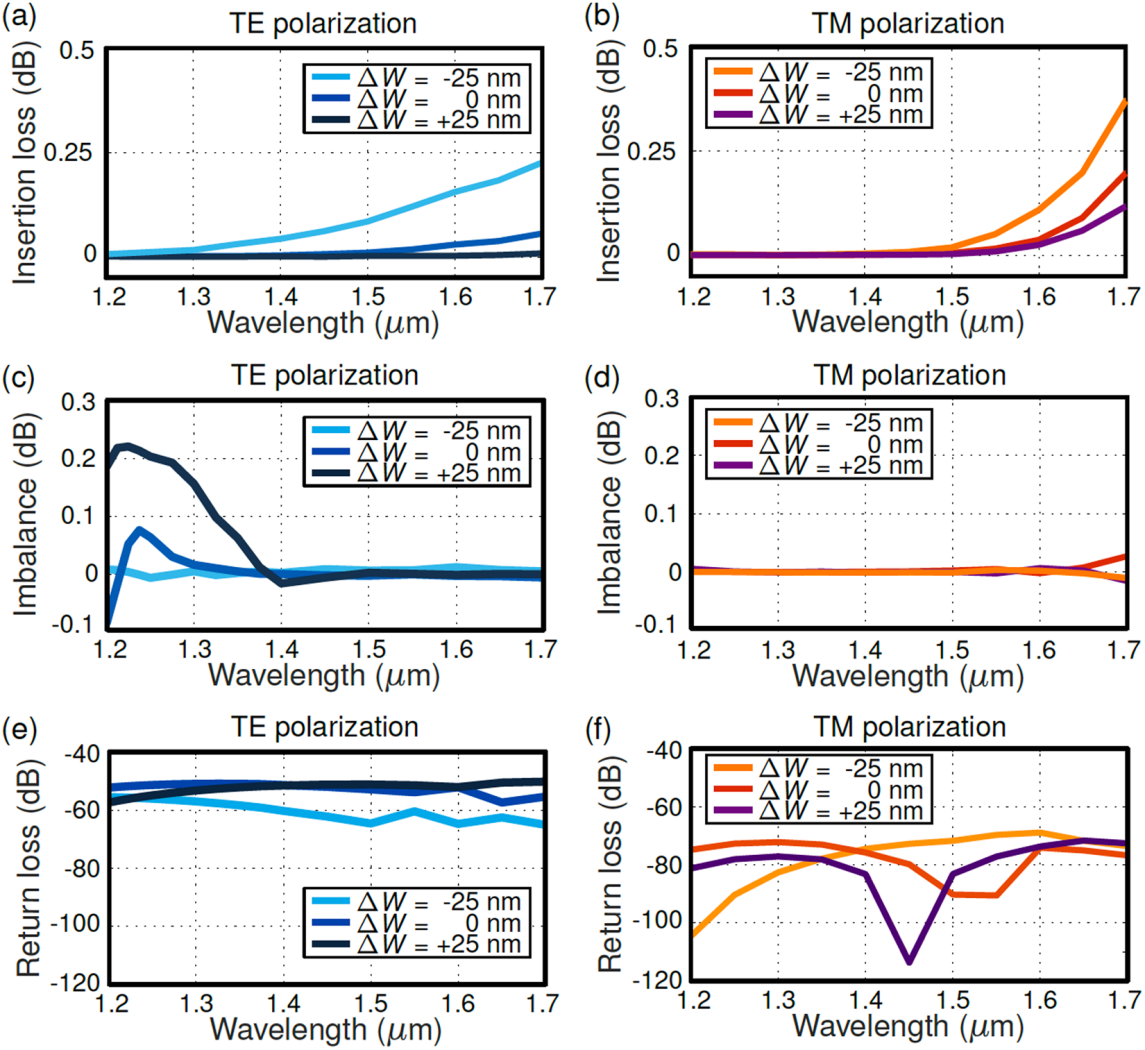
Insertion loss for TE (**a**) and TM (**b**) polarizations as a function of wavelength for the nominal design. Imbalance is also shown for TE (**c**) and TM (**d**) polarizations. Return loss calculated for TE (**e**) and TM (**f**) polarizations. We considered the nominal design (∆*W* = 0 nm) and deviations in all waveguide widths of ∆*W*  = ±25 nm.

The performance and robustness against fabrication errors of the optimized device were studied via 3D EME simulations^47^. The effects of material dispersion were included in the analysis for Si and SiO_2_ layers. As the main figures of merit, we considered the insertion loss (IL), defined as the amount of power relative to the input power that is not transferred to any output; the imbalance (IB), defined as the power difference between the two output ports relative to the input power; and the return loss (RL), defined as the ration between back reflections and input power. These three figures of merit were calculated for the complete splitter (sections I, II and III) considering the nominal design and ±25 nm deviations in the waveguide width. In order to mimic typical fabrication errors produced by under- and over-etching, the central position of each waveguide is maintained during the tolerance study, widening/narrowing them at a fixed ratio. As a consequence, the symmetry of the structure is maintained, whereas waveguide width and gap size are altered in a factor of Δ*W*. Figure 3 shows the simulation results. An IL below 0.5 dB, an imbalance below 0.3 dB, and RL better than −40 dB in the 1200–1700 nm wavelength range are predicted for both the nominal and biased designs, confirming the resilience of the device against fabrication errors. It should be noted that at shorter wavelengths the strip-to-slot transition may start guiding higher order TE modes, resulting in slight power imbalance, below 0.1 dB for the nominal design. This effect is accentuated when increasing the waveguide width (Δ*W* =+25 nm) and mitigated when reducing the waveguide width (Δ*W* = −25 nm). Conversely, insertion losses decrease by increasing the waveguide width. Therefore, our nominal design is a compromise between low insertion losses, flat imbalance and tolerances to fabrication errors.

**Figure 4:**
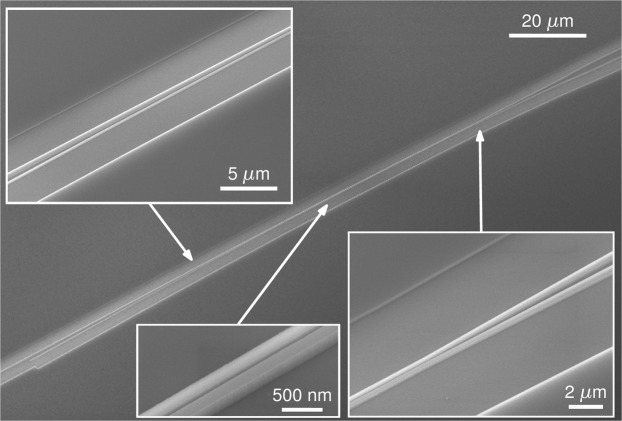
Scanning electron microscope images of the fabricated splitter. Insets (left to right): strip-to-slot mode converter (section I), central slot waveguide (section II) and slot-to-strip output taper (section III).

Figure 4 shows scanning electron microscope (SEM) images of the fabricated beam splitter before PMMA deposition (see the Methods section). The splitter was characterized using both a Mach-Zehnder interferometer (MZI) and cascaded stages using the experimental setup described in the Methods section. The MZI configuration allows precise estimation of the imbalance, extracted from measured extinction ratio (ER)^28,48^. However, insertion loss estimation from the MZI response is less accurate, as it inherently combines both MZI loss and chip-coupling loss. Given the low loss of our device, total insertion loss measured at the MZI is strongly affected by the non-uniformity of the chip-coupling loss, e.g. due to non-perfect cleaving of the facets. To overcome this limitation, insertion loss was accurately measured by characterizing the response of cascaded splitters^32^.

**Figure 5:**
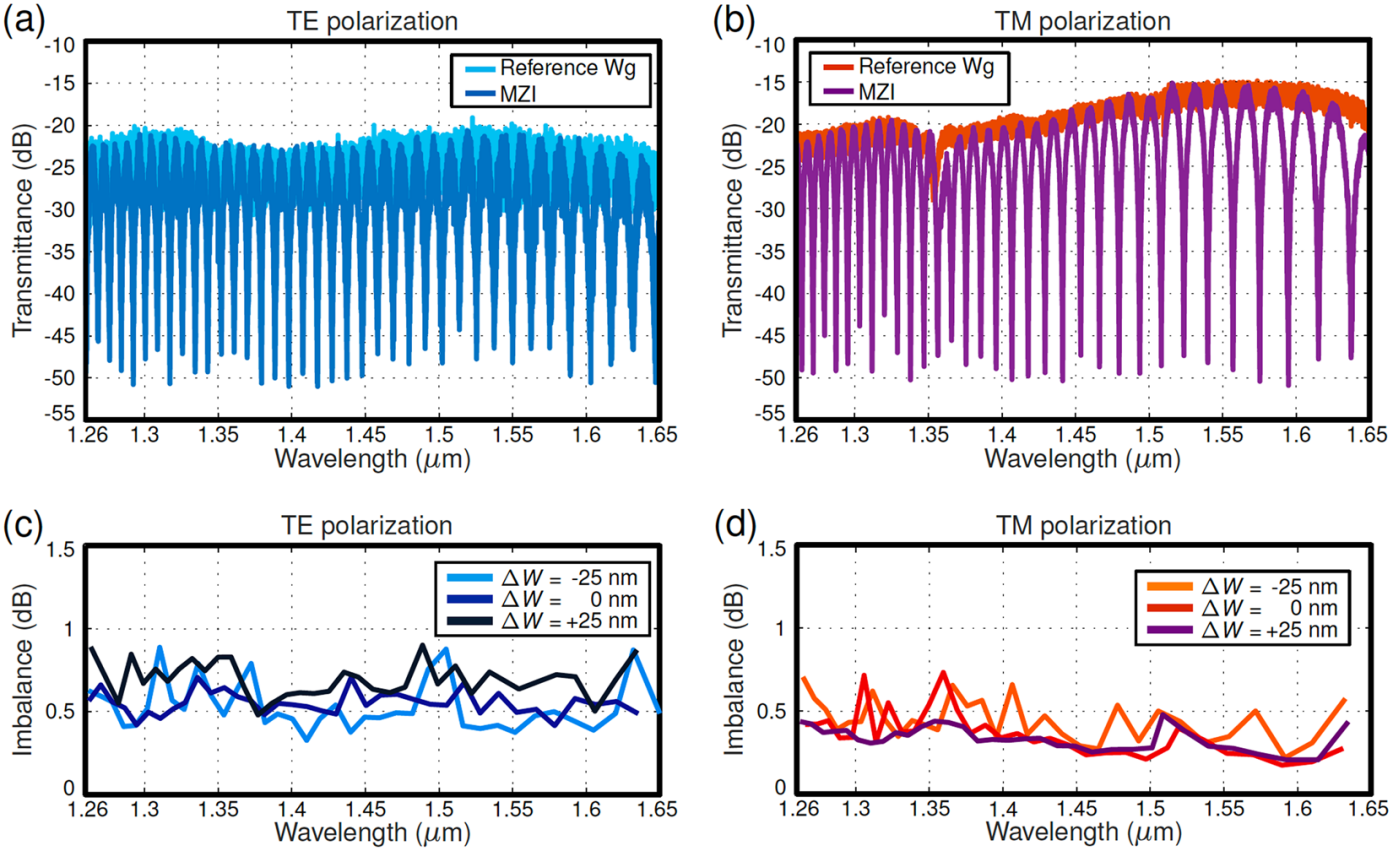
Measured spectra of a Mach-Zehnder interferometer with back-to-back beam splitters, compared to a reference strip waveguide for TE (**a**) and TM (**b**) polarizations. All spectra are normalized to calibrate out setup loss. Imbalance extracted from measured ER in MZI with nominal and ±25 nm biased splitters for (**c**) TE and (**d**) TM polarizations.

In the MZI configuration, two identical splitters were connected in a back-to-back configuration with an arm imbalance of 40 *µ*m. Figure 5(a,b) show the measured transmittance spectra of the MZI interferometer, comprising two nominal splitters, for both the TE and TM polarizations compared to a reference strip waveguide. The device showed measured extinction ratios (ERs) higher than 20 dB and 25 dB over a 390 nm wavelength range (1.26–1.65 *µ*m) for the TE and TM polarizations, respectively. The imbalance was calculated from the measured ER^28,48^ for MZIs comprising nominal design and waveguide width variations of ±25 nm (see Fig. 5(c,d)). Nominal and biased designs yield less than 0.9 dB in the full wavelength range for both polarizations, illustrating the robustness of the proposed approach.

**Figure 6:**
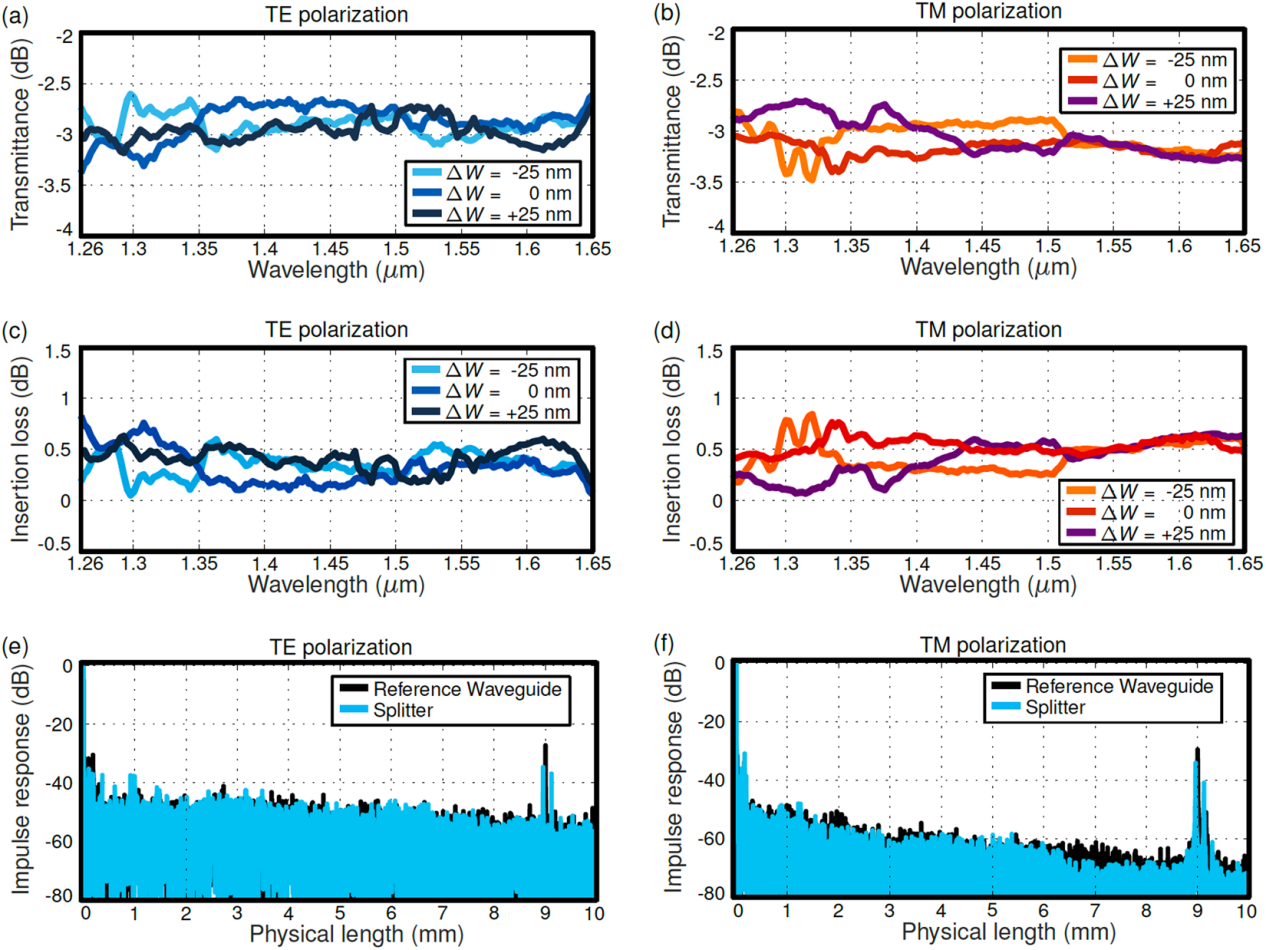
Transmittance of splitters for (**a**) TE and (**b**) TM polarizations, measured through linear regression of the response of five cascaded stages. Insertion loss for (**c**) TE and (**d**) TM polarizations obtained from transmittance of five cascaded splitters and taking into account the worst-case imbalance from the MZI experiment. Measured optical reflection signals in spatial domain of a nominal splitter for (**e**) TE and (**f**) TM polarizations, compared to those of a reference waveguide. The sample is 9 mm long, and the splitter is placed 2 mm away from the output facet.

Five beam splitter stages were cascaded to carry out an accurate measurement of the transmission spectrum of the device. We included both the nominal design and biased structures with waveguide width variations of ±25 nm, allowing us to determine the tolerance to fabrication errors. One output of each splitter stage was collected at the chip facet, whereas the second output was directed to the next splitter stage, enabling transmission measurement through the linear regression fitting of the five resulting signals. As shown in Fig. 6, the slot-based splitters exhibited a deviation of less than ±0.5 dB with respect to the ideal −3 dB transmission for both polarizations within the 1260–1650 nm wavelength range. The same robust performance was demonstrated even when waveguide width variations of up to ±25 nm were introduced. Simulation analysis predicted deviations below 0.25 dB. This small discrepancy is attributed to fabrication imperfections such as waveguide roughness. Insertion loss of the proposed beam splitter is obtained from the transmittance of the cascaded splitters and taking into account the worst-case imbalance from the MZI experiment. As shown in Fig. 6(c,d), our device yields insertion loss below 1 dB in a 390 nm bandwidth, for both polarizations, even in the presence of fabrication errors as large as ±25 nm. Finally, back-reflections in the coupler are studied experimentally by transforming the output signal of the chip into the spatial domain through the minimum phase technique^49^. This process was performed for both the splitter and a reference waveguide, showing no evidence of increased back-reflections caused by the splitter for neither TE nor TM polarizations (Fig. 6(e,f)). Note that the peaks shown near 0 mm and 9 mm correspond to direct transmission and reflection in the input facet, respectively, whereas any back-reflection caused by the splitter would appear as a peak in the region near 7 mm. The absence of this latter peak is in good agreement with the low back-reflection predicted by calculations.

## Discussion

In conclusion, we have proposed and experimentally demonstrated an ultra-broadband and polarization-independent optical beam splitter based on a single-mode slot waveguide with a symmetric slot-to-strip transition. The single-mode operation of the slotted section prevents wavelength-dependent mode beating, which fundamentally limits the bandwidths of conventional DCs and MMIs, while the symmetric geometry ensures equal power splitting for both the TE and TM polarizations even with comparatively large fabrication errors. The splitter yields a near-ideal transmission of −3 ± 0.5 dB for both polarizations in an unprecedented wavelength bandwidth of 390 nm, covering the O, E, S, C and L telecommunication bands. This outstanding performance is maintained in the presence of fabrication deviations as large as ±25 nm, thereby relaxing fabrication constraints. We believe that the proposed optical beam splitter has excellent potential for use in the next generation of photonic integrated circuits as a key building block enabling dual-polarization and ultra-broadband silicon photonics devices.

## Methods

### Device fabrication and experimental characterization

The beam splitter was fabricated in SOI wafers with a 220-nm-thick silicon and a 2 *µ*m BOX layer. Electron beam lithography (Nanobeam NB-4 system, 80 kV) was used to define the splitter pattern. A dry etching process with an inductively coupled plasma etcher (SF6 gas) was employed to transfer the pattern to the silicon layer. Finally, 1-*µ*m-thick PMMA was deposited as the upper cladding layer.

Devices were characterized using three tunable lasers, covering sequentially the 1260 nm to 1650 nm wavelength range. Light was injected into the chip via 3-*µ*m-wide waveguide edge couplers, using a lensed polarization-maintaining fiber, after polarization selection with a rotating quarter-waveplate and a linear polarizer. Light coming out of the chip was directed through a microscope objective and a linear polarizer to a photodetector.

## Data Availability

The data generated during and/or analysed during the current study are available from the corresponding author on reasonable request.

## References

[1] Reed GT, Mashanovich G, Gardes FY, Thomson DJ (2010). Silicon optical modulators. Nat. Photonics.

[2] Zheng X (2011). Ultra-efficient 10Gb/s hybrid integrated silicon photonics transmitter and receiver. Opt. Express.

[3] Dong P (2014). Monolithic silicon photonic integrated circuits for compact 100 + Gb/s coherent optical receivers and transmitters. IEEE J. Sel. Top. Quantum Electron..

[4] Vacondio F (2010). A silicon modulator enabling RF over fiber for 802.11 OFDM signals. IEEE J. Sel. Top. Quantum Electron..

[5] Chow CW, Yeh CH, Lo SM, Li C, Tsang HK (2011). Long-reach radio-over-fiber signal distribution using single-sideband signal generated by a silicon-modulator. Opt. Express.

[6] De Vos K, Bartolozzi I, Schacht E, Bienstman P, Baets R (2007). Silicon-on-insulator microring resonator for sensitive and label-free biosensing. Opt. Express.

[7] Estevez MC, Alvarez M, Lechuga LM (2012). Integrated optical devices for lab-on-a-chip biosensing applications. Laser Photonics Rev..

[8] Poulton CV (2017). Coherent solid-state LIDAR with silicon photonic optical phased arrays. Opt. Lett..

[9] Redding B, Liew SF, Sarma R, Cao H (2013). Compact spectrometer based on a disordered photonic chip. Nat. Photonics.

[10] Velasco AV (2013). High-resolution Fourier-transform spectrometer chip with microphotonic silicon spiral waveguides. Opt. Lett..

[11] Koos C (2009). All-optical high-speed signal processing with silicon-organic hybrid slot waveguides. Nat. Photonics.

[12] Jacobsen RS (2006). Strained silicon as a new electro-optic material. Nature.

[13] Chen X, Liu W, Zhang Y, Shi Y (2017). Polarization-insensitive broadband 2x2 3 dB power splitter based on silicon-bent directional couplers. Opt. Lett..

[14] Offrein BJ, Bona GL, Germann R, Massarek I, Erni D (1998). A very short planar silica spot-size converter using nonperiodic segmented waveguide. J. Light. Technol..

[15] Cheben P, Xu DX, Janz S, Densmore A (2006). Subwavelength waveguide grating for mode conversion and light coupling in integrated optics. Opt. Express.

[16] Cheben P (2010). Refractive index engineering with subwavelength gratings for efficient microphotonic couplers and planar waveguide multiplexers. Opt. Lett..

[17] Marcatili EAJ (1969). Dielectric rectangular waveguide and directional coupler for integrated optics. Bell Syst. Tech. J..

[18] Trinh PD, Yegnanarayanan S, Jalali B (1995). Integrated optical directional couplers in silicon-on-insulator. Electron. Lett..

[19] Yamada H, Chu T, Ishida S, Arakawa Y (2005). Optical directional coupler based on Si-wire waveguides. IEEE Photonics Technol. Lett..

[20] Xing J (2013). Silicon-on-insulator-based adiabatic splitter with simultaneous tapering of velocity and coupling. Opt. Lett..

[21] Cong GW (2014). Demonstration of a 3-dB directional coupler with enhanced robustness to gap variations for silicon wire waveguides. Opt. Express.

[22] Yun, H. *et al*. 2x2 broadband adiabatic 3-dB couplers on SOI strip waveguides for TE and TM modes. *In CLEO: Sci*. *Innov*. STh1F–8 (2015).

[23] Xu, L. *et al*. Polarization independent adiabatic 3-dB coupler for silicon-on-insulator. *In CLEO: Sci*. *Innov*. SF1I–5 (2017).

[24] Tamazin, H. *et al*. Ultra-broadband compact adiabatic coupler in silicon-on-insulator for joint operation in the C- and O-bands. *In CLEO: Sci*. *Innov*. STh4B–4 (2018).

[25] Yun H, Chrostowski L, Jaeger NA (2018). Ultra-broadband 2x2 adiabatic 3 dB coupler using subwavelength-grating-assisted silicon-on-insulator strip waveguides. Opt. Lett..

[26] Yun H (2016). Broadband 2x2 adiabatic 3 dB coupler using silicon-on-insulator sub-wavelength grating waveguides. Opt. Lett..

[27] Halir R (2012). Colorless directional coupler with dispersion engineered sub-wavelength structure. Opt. Express.

[28] Wang Y (2016). Compact broadband directional couplers using subwavelength gratings. IEEE Photonics J..

[29] Yajima H (1973). Dielectric thin-film optical branching waveguide. Appl. Phys. Lett..

[30] Burns W, Milton A (1975). Mode conversion in planar-dielectric separating waveguides. IEEE J. Quantum Electron..

[31] Rickman AG, Reed GT (1994). Silicon-on-insulator optical rib waveguides: loss, mode characteristics, bends and y-junctions. IEE P-Optoelectron..

[32] Zhang Y (2013). A compact and low loss Y-junction for submicron silicon waveguide. Opt. Express.

[33] Sakai A, Fukazawa T, Baba T (2002). Low loss ultra-small branches in a silicon photonic wire waveguide. IEICE Trans. Electron..

[34] Wang Y, Gao S, Wang K, Skafidas E (2016). Ultra-broadband and low-loss 3 dB optical power splitter based on adiabatic tapered silicon waveguides. Opt. Lett..

[35] Soldano LB, Pennings EC (1995). Optical multi-mode interference devices based on self-imaging: principles and applications. J. Light. Technol..

[36] Maese-Novo A (2013). Wavelength independent multimode interference coupler. Opt. Express.

[37] Halir R (2016). Ultra-broadband nanophotonic beamsplitter using an anisotropic sub-wavelength metamaterial. Laser Photonics Rev..

[38] Li X (2013). Compact and low-loss silicon power splitter based on inverse tapers. Opt. Lett..

[39] Rasigade G, Le Roux X, Marris-Morini D, Cassan E, Vivien L (2010). Compact wavelength-insensitive fabrication-tolerant silicon-on-insulator beam splitter. Opt. Lett..

[40] Han L, Kuo BPP, Alic N, Radic S (2018). Ultra-broadband multimode 3 dB optical power splitter using an adiabatic coupler and a Y-branch. Opt. Express.

[41] Frandsen LH (2004). Ultralow-loss 3-dB photonic crystal waveguide splitter. Opt. Lett..

[42] Feng NN, Sun R, Kimerling LC, Michel J (2007). Lossless strip-to-slot waveguide transformer. Opt. Lett..

[43] Palmer R (2013). Low-loss silicon strip-to-slot mode converters. IEEE Photonics J..

[44] Xu Q, Almeida VR, Panepucci RR, Lipson M (2004). Experimental demonstration of guiding and confining light in nanometer-size low-refractive-index material. Opt. Lett..

[45] Almeida VR, Xu Q, Barrios CA, Lipson M (2004). Guiding and confining light in void nanostructure. Opt. Lett..

[46] Palik, E. D. Handbook of optical constants of solids (Academic press, 1998).

[47] MODE Solutions, Lumerical Solutions Inc. http://www.lumerical.com/.

[48] Bogaerts W (2012). Silicon microring resonators. Laser Photonics Rev..

[49] Halir R (2009). Characterization of integrated photonic devices with minimum phase technique. Opt. Express.

